# Expression of TRAIL, IP-10, and CRP in children with suspected COVID-19 and real-life impact of a computational signature on clinical decision-making: a prospective cohort study

**DOI:** 10.1007/s15010-023-01993-1

**Published:** 2023-02-09

**Authors:** Franziska Fröhlich, Benjamin Gronwald, Johannes Bay, Arne Simon, Martin Poryo, Jürgen Geisel, Sina A. Tegethoff, Katharina Last, Jürgen Rissland, Sigrun Smola, Sören L. Becker, Michael Zemlin, Sascha Meyer, Cihan Papan

**Affiliations:** 1 Center for Infectious Diseases, Institute of Medical Microbiology and Hygiene, Saarland University, Kirrberger Strasse, Building 43, Homburg, Germany; 2 Department of General Pediatrics and Neonatology, Saarland University Hospital, Homburg, Germany; 3 Department of Pediatric Hematology and Oncology, Saarland University Hospital, Homburg, Germany; 4 Department of Pediatric Cardiology, Saarland University Hospital, Homburg, Germany; 5 Department of Clinical Chemistry and Laboratory Medicine, Saarland University Medical Centre, Saarland University Hospital, Homburg, Germany; 6 Institute for Hygiene and Public Health, University Hospital Bonn, Bonn, Germany; 7 Institute of Virology, Saarland University Medical Center, Homburg, Germany; 8 Helmholtz Center for Infection Research (HZI), Helmholtz Institute for Pharmaceutical Research Saarland (HIPS), Saarland University Campus, Saarbrücken, Germany

**Keywords:** COVID-19, Antimicrobial stewardship, Children, Emergency department, Clinical decision-making, Biomarkers, C-reactive protein, TRAIL protein, IP-10 protein

## Abstract

**Purpose:**

We evaluated the host-response marker score “BV” and its components TRAIL, IP-10, and CRP in SARS-CoV-2 positive children, and estimated the potential impact on clinical decision-making.

**Methods:**

We prospectively analyzed levels of TRAIL, IP-10, CRP, and the BV score, in children with suspected COVID-19. Classification of infectious etiology was performed by an expert panel. We used a 5-point-questionnaire to evaluate the intention to treat with antibiotics before and after receiving test results.

**Results:**

We screened 111 children, of whom 6 (5.4%) were positive for SARS-CoV-2. A total of 53 children were included for the exploratory analysis. Median age was 3.1 years (interquartile range [IQR] 1.3–4.3), and 54.7% (*n* = 29) were girls. A viral and a bacterial biomarker pattern was found in 27/53 (50.9%) and 15/53 (28.3%), respectively. BV scores differed between COVID-19, children with other viral infections, and children with bacterial infections (medians 29.5 vs. 9 vs. 66; *p* = 0.0006). Similarly, median TRAIL levels were different (65.5 vs. 110 vs. 78; *p* = 0.037). We found no differences in IP-10 levels (555 vs. 504 vs. 285; *p* = 0.22). We found a concordance between physicians’ “unlikely intention to treat” children with a viral test result in most cases (*n* = 19/24, 79.2%). When physicians expressed a “likely intention to treat” (*n* = 15), BV test revealed 5 bacterial, viral, and equivocal scores each. Antibiotics were withheld in three cases (20%). Overall, 27/42 (64%) of pediatricians appraised the BV test positively, and considered it helpful in clinical practice.

**Conclusion:**

Host-response based categorization of infectious diseases might help to overcome diagnostic uncertainty, support clinical decision-making and reduce unnecessary antibiotic treatment.

## Introduction

Over- and misuse of antibiotics is a major concern in pediatric cohorts and drives emergence of antimicrobial resistance (AMR) as well as individual side effects, including alterations of the microbiome [1–3]. Prudent antibiotic use is promoted by knowledge of whether an infection is present, as well as identification of its etiology [4]. However, diagnostic uncertainty in particular concerning the question whether the infection is viral or bacterial in origin, remains high among pediatric practitioners, especially when diagnosis is based solely on clinical judgment [5]. This challenge has persisted throughout the COVID-19 pandemic [6, 7], with clinical manifestations of pediatric SARS-CoV-2 infection ranging from upper respiratory infection to severe inflammatory states, including multisystem inflammatory syndrome in children (MIS-C) [8]. In addition, point-of-care screening tools such as rapid SARS-CoV-2 antigen tests have yielded low sensitivities for children [9].

Several studies have shown that host-protein biomarkers are promising tools for both detection and differentiation of bacterial and viral infections [10, 11]. A novel immunoassay combines measurements of TNF-related apoptosis-inducing ligand (TRAIL), interferon gamma-induced protein-10 (IP-10), and C-reactive protein (CRP) to generate a score (BV). With a negative predictive value of 98.9%, the score has promise to be valuable in reducing antibiotic overuse in viral infections [12]. In addition, newer data suggest that TRAIL correlates with clinical severity in children with respiratory tract infections [13]. Of note, studies on other biomarker combinations, e.g., CRP and myxovirus resistance protein A, have shown promising results in regard to screening for COVID-19 and triaging in the emergency department (ED) [14, 15].

Here, we (i) analyzed the BV score and its three constituent biomarkers in SARS-CoV-2 infected children, in comparison with other viral and bacterial infections, presenting to a University Medical Center; this is a subgroup analysis of the previously reported DIRECTOR study [16].

Furthermore, we (ii) evaluated the BV score as an additional tool in the pediatric ED as an exploratory endpoint, and report on the physicians’ assessments of the associated potential for improvement in clinical practice, particularly in antibiotic prescribing.

## Methods

Between November 2020 and August 2021, children and adolescents aged > 90 days with symptoms of a respiratory tract infection or fever without an apparent focus, compatible with COVID-19, were recruited at Saarland University Children’s Hospital in Homburg, Germany. The study was approved by the Ethics committee of the Ärztekammer des Saarlandes (reference number 019/20).

As standard of care, every patient received a nasopharyngeal swab which was tested via polymerase chain reaction for the presence of SARS-CoV-2. In addition, a multiplex PCR was performed to test for several respiratory viruses and bacteria. The multiplex panel (FTD Respiratory pathogens 21, Siemens Healthineers) included the following pathogen detections: influenza A; influenza A/H1N1; influenza B; parainfluenza types 1, 2, 3, 4; coronavirus NL63, 229E, OC43, HKU1; human metapneumovirus (A/B); human bocavirus; rhinovirus; adenovirus; respiratory syncytial virus (A/B); parechovirus; enterovirus and *Mycoplasma pneumoniae*. This commercial, real-time PCR assay was performed according to the manufacturer’s instructions and as previously described [17]. Bacterial infections were confirmed by bacteriologic cultures of blood, throat swabs, or other clinical samples.

According to the results and in due consideration of all patient data, we divided the cohort into three groups: patients with COVID-19, patients with other viral respiratory tract infection, and patients with bacterial infection.

We measured TRAIL, IP-10 and CRP and calculated the corresponding BV score by placing 100 µL of patients’ serum in a MeMed BV^®^ cartridge and running the test on MeMed Key^®^ (MeMed; Tirat Carmel, Israel) as described previously [16, 18]. For this purpose, an additional serum tube was collected during routinely performed blood draws. The resulting BV score ranges between 0 and 100 and defines infectious etiology as viral (0–34), equivocal (35–65) or bacterial (66–100). We compared the BV score’s alignment with infectious disease etiology in comparison with CRP, for which we used CRP bins of < 20 mg/L (indicating viral etiology), 20–80 mg/L (inconclusive), and > 80 mg/L (bacterial), based on previous literature [19]. For statistical analyses, we used Kruskal–Wallis tests and pair-wise Wilcoxon rank sum tests with Bonferroni correction, using RStudio (Version 2022.07.1 + 554).

For the exploratory endpoint, we handed a 5-point questionnaire to ED physicians, which contained questions about their intention to initiate antibiotic therapy (before and after receiving test results); furthermore, we asked physicians if the test result, which was accessible to the physician, influenced their clinical decision-making.

Three pediatricians, each with more than 10 years of work experience, formed an expert panel and evaluated each patient’s disease etiology (before and after receiving test results) while blinded for each other’s adjudication, as described previously [12]. Majority voting of the expert panel was used as additional classification. Of note, none of the experts was involved in the patients’ care.

## Results

### Study population

Out of 111 children screened in our pediatric ED for possible SARS-CoV-2 infection, 6 children (5.4%) tested positive for SARS-CoV-2 (COVID-19 group), one of whom was diagnosed with MIS-C. The median age of the COVID-19 group was 9.3 years (IQR 4–13.2), and 50% (*n* = 3) were girls. Among the 111 patients, 53 fell within the original instructions for use of the BV. The median age of this exploratory study cohort (*n* = 53) was 3.1 years (interquartile range [IQR] 1.3–4.3) and 29 (54.7%) were girls. The viral (non-COVID-19) group consisted of 27 children (50.9%), in which 15 were girls (55.6%) and the median age was 1.7 years (IQR 1.2–3.3). Bacterial infection (bacterial group) was diagnosed in 15 patients (28.3%) with a median age of 3.5 years (IQR 1.2–4.5), including 9 girls (60%).

### Results of biomarker measurements

In the COVID-19 group, there was a median BV score of 29.5 (IQR 8–41.3) and a median TRAIL level of 65.5 pg/mL (IQR 42–167) (Table 1). IP-10 levels showed a median of 555 pg/mL (IQR 336–2117.3) and CRP levels reached a median of 19.5 mg/L (IQR 2–42.2). The child with MIS-C (with positive SARS-CoV-2 PCR) showed a BV score of 100. The TRAIL level was 19 pg/mL, IP-10 yielded a level of higher than 6000 pg/mL and the value of CRP was 196.9 mg/L (Table 1).

**Table 1:** Demographic and biomarker data of the COVID-19 cases within the cohort; age in years; F: female, M: male

	Age	Sex	Test IFU fulfilled	BV score	TRAIL (pg/mL)	CRP (mg/L)	IP-10 (pg/mL)	Comment
KK005	6.9	F	Yes	100	19	196.9	6001	MIS-C
KK012	3.1	M	Yes	1	200	6.3	320	
KK024	2.5	F	No	0	301	0.5	726	
KK026	22.9	F	No	29	35	0.5	99	
KK035	13.7	M	Yes	45	68	45.7	384	
KK062	11.6	M	No	30	63	32.6	2581	

The median BV score in the viral (non-COVID-19) group was 9 (IQR 0–25.5), whereas the median levels for the individual biomarkers were as follows: TRAIL 110 pg/mL (IQR 79.5–245.5), IP-10 504 pg/mL (IQR 316.5–813), and CRP 12.3 mg/L (IQR 3.7–26.9).

**Fig. 1:**
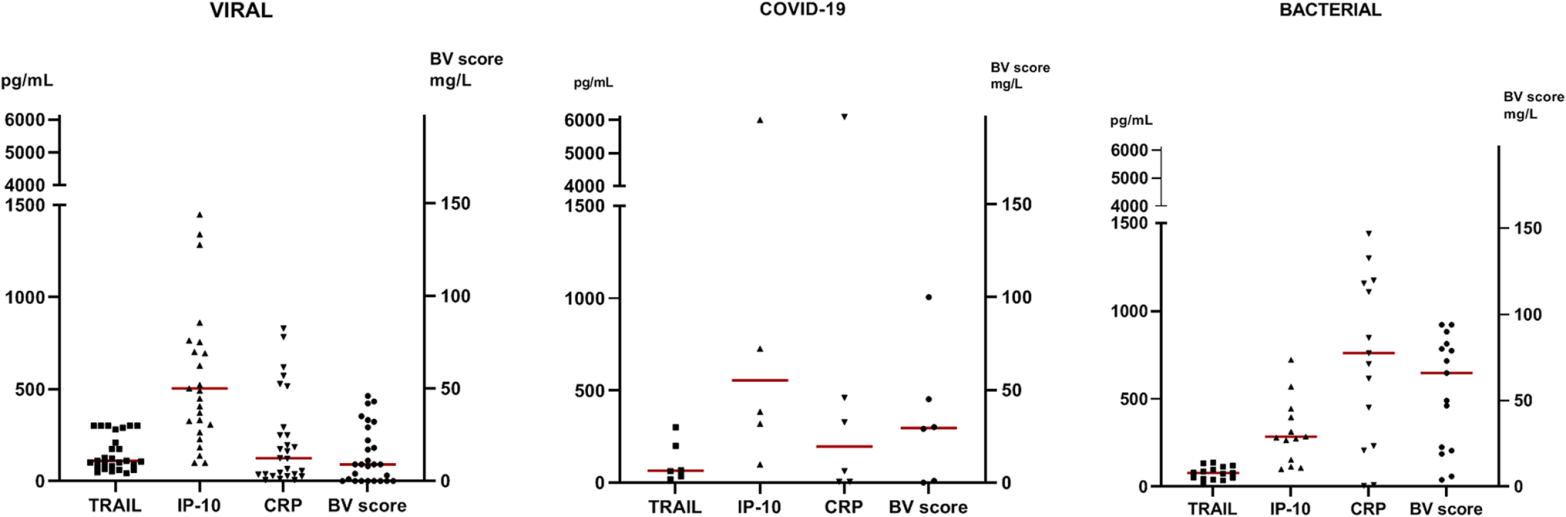
Comparison of TRAIL, IP-10, CRP and BV score, among the three subgroups (median marked with red line)

Patients with bacterial infections yielded a median score of 66 (IQR 22–81.5). The median measurements for TRAIL, IP-10 and CRP were 78 pg/mL (IQR 48–105.5), 285 pg/mL (IQR 209–507), and 82.8 mg/L (IQR 34.9–119), respectively (Fig. 1).

BV scores differed significantly between the three groups COVID-19, children with other viral infections, and children with bacterial infections (*p* = 0.0006). The difference was significant in pair-wise comparison for viral vs. bacterial groups (*p* = 0.0003), while there was no difference between COVID-19 and the viral or bacterial groups (*p* = 0.69 and *p* = 0.78). Similarly, median TRAIL levels were significantly different over all groups (*p* = 0.037), with the difference between viral and bacterial being significant (*p* = 0.035), and no difference between COVID-19 and the two other groups (*p* = 0.67 and *p* = 1.0). We found no significant differences in IP-10 levels (*p* = 0.22).

**Fig. 2:**
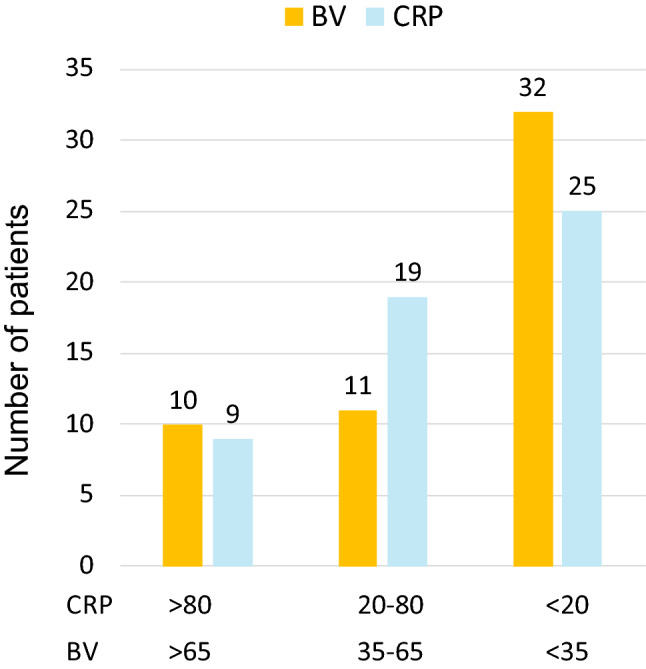
Comparison of categorizing disease etiology by BV and CRP (mg/L); BV 0–34: viral etiology, BV 35–65 equivocal etiology, BV 66–100 bacterial etiology; *n* = 53

When comparing the BV score to CRP levels to categorize diseases’ etiology, the score indicated an equivocal result in 11 cases, whereas inconclusive levels of CRP (20–80 mg/L) appeared in 19 patients (Fig. 2).

### Potential impact of the BV score on clinical decision-making

**Fig. 3:**
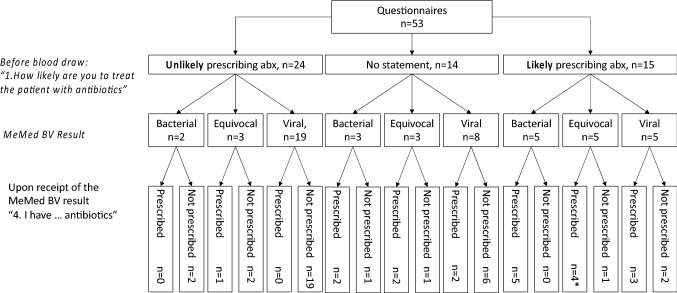
Intention to prescribe antibiotics before and after BV result (* already treated with antibiotics, prescribed by family pediatrician); *abx* antibiotics

After initial physical examination, ED physicians stated that they were unlikely to treat the patients with antibiotics in 24/53 cases (45.3%). Among these patients, the BV score indicated a bacterial infectious etiology for 2/24 (8.3%) and a viral etiology for 19/24 (79.2%) of the patients. Three of the 24 cases yielded equivocal BV scores. Upon receiving these results, the physicians changed their mind regarding one patient (equivocal) and initiated an antibiotic therapy (Fig. 3).

Although ED doctors initially did not intend to prescribe antibiotics for the two children with bacterial BV scores, antibiotics were prescribed during their hospital stay. Experts considered those cases to be of viral and non-infectious origin. Overall, our expert panel aligned with the score’s result in 17 out of 24 cases (70.8%).

When physicians stated they were uncertain whether to prescribe antibiotics after initial examination (“no statement”, *n* = 14), they did not prescribe antibiotics in 6/8 cases with a viral BV result. A bacterial result (*n* = 3, 21.4%) prompted them to consider antibiotic therapy in two cases (Fig. 3). Pediatricians refused to prescribe antibiotics for one bacterial case, in whom *Campylobacter* spp. was detected in stool. Experts assumed viral origin in this case. Across all 14 ‘no statement’ cases, our experts identified 9 viral infections and one bacterial infection. One case was considered to be indeterminable. There was no agreement for 3 cases. After initial physical examination, ED physicians stated that they were likely to prescribe antibiotics for 15/53 patients (28.3%). The BV score indicated a bacterial, equivocal, and viral origin in an equal measure in this group (*n* = 5, respectively). Knowledge of the score resulted in withholding of antibiotics in 3/15 (20%) cases (Fig. 3). A follow-up interview did not reveal any unfavorable outcome for those three patients, and they all did well after being discharged. Experts and medical files confirmed the viral results in two cases and notably, antibiotics were not prescribed. However, for one patient, antibiotic treatment became necessary during their hospital stay. (Fig. 3; Table 2).

**Table 2:** Intention and real-life antibiotic prescription during hospital stay: demographics, pediatricians’ and experts’ assessment compared to BV result and lab data; age in years

Age	Sex	Days from symptom onset	Questionnaire: Intent to Prescribe Abx before test result	BV	CRP	WBC	Questionnaire: intent to prescribe Abx after test result	Real-life data: Abx prescribed (Medical file)	Microbiology	Discharge diagnosis	Experts’ vote
1.6	F	2	Probable	9	12.2	6.9	Not prescribed	–	Urine culture: *Proteus* *mirabilis*, < 10^3/mL	Febrile upper respiratory tract infection	Viral
1	M	0	Probable	0	2.9	11.5	Not prescribed	–	Nasopharyngeal swab: Coronavirus NL63	Febrile upper respiratory tract infection	Viral
2.3	F	1	No statement	66	72.3	10.1	Not prescribed	–	Stool: *Campylobacter jejuni*/*coli*	1st febrile seizure, gastroenteritis [Bacterial etiology]	Viral
3.4	F	1	No statement	29	78.7	13.6	Prescribed (and then canceled)	Yes	–	Suspected periodic fever, suspected febrile seizure	No agreement
4.4	M	3	No statement	33	14.3	9.7	Prescribed	Yes	Nasopharyngeal swab: *Streptococcus* *pneumoniae* RSV	first day: presented under antibiotics, positive rapid strep-test; third day: negative culture; fourth day: Abx stopped, bronchitis	Viral
6.9	F	4	Unlikely	100	198.8	12.5	Not prescribed	Yes	Throat swab: *Staphylococcus* *aureus*	MISC: Multisystem Inflammatory Syndrome in Children, cardiac involvement after SARS-CoV-2 infection	Non-infectious
5	F	2–3	Very unlikely	78	12.9	19.6	Not prescribed	Yes	Urine culture – Positive, CFU < 10^3/mL	Rhabdomyolysis, suspected myocarditis [Indeterminate etiology]	Viral

*Abx *antibiotics, *CFU* colony-forming units, *CRP* C-reactive protein (mg/L), *WBC* white blood count (× 10^9^/L)

Overall, experts aligned with BV results in 9/15 cases (64.3%). Of note, when assessing the entire hospital stay of the 53 patients, antibiotic treatment rates were higher than the rate derived from the physician’s responses via questionnaire (47.2% prescriptions in total cohort, *n* = 25). Based on their responses in the questionnaire, 27 of the 42 treating physicians (64%) considered the BV score to be a helpful tool in clinical practice: this includes confirming their treatment decision (*n* = 22, 52%) as well as changing therapy regimen (12%). In 15 instances (36%), pediatricians reported that the tool was no help in clinical decision-making. This impression of the BV test’s utility was mostly shared by our expert panel: positive feedback (confirming decisions, changing therapy regimen) was stated for 67.9%, 45.3%, and 69.8% of the cases by the three senior physicians, respectively.

## Discussion

In this study, we showed no statistically significant difference in host-response biomarker expressions and the BV score for children with SARS-CoV-2 infections as compared to children with other viral or bacterial infections, mainly due to the small sample size. Of note, our cohort included a patient with MIS-C, whose particular biomarker expression was most likely caused by an immune dysregulation reminiscent of critical adult COVID-19 [16].

Although there is only one case described, testing for MIS-C via host-response biomarkers may pose a promising tool: diagnosing MIS-C can be challenging and is mostly based on exclusion of other diseases [20, 21]. Revealing MIS-C via blood test and biomarker constellation might shorten the time to diagnosis.

We observed that utilization of the BV score in a pediatric ED guides clinical decision-making and improves the appropriate use of antibiotics. As it was mentioned by pediatricians themselves, the tool offers guidance and might help to overcome diagnostic uncertainty. The low turnaround time of one hour also contributes to a short time to treatment, which is associated with lower mortality [22].

Interestingly, BV scores were less often equivocal than CRP yielded inconclusive values between 20 and 80 mg/L. This is of particular importance, since the clinical utility of CRP in particular in an early stage of the infection is often hampered by this area of uncertainty in which CRP offers inferior diagnostic accuracy with respect to detecting serious bacterial infections in children [19].

Knowledge of the score’s result led to rethinking among practitioners and lowered prescription rates when the test revealed a viral infectious etiology. The fact that our expert panel’s opinion was aligned to the score’s group assignment in most cases supports its diagnostic accuracy. These real-world data support the clinical value of the BV test when there is diagnostic uncertainty at the patient’s first assessment.

Both, practitioners and experts, reported very positively about the use of the test, especially in the dilemma of diagnostic uncertainty, where confirming results are desperately needed.

Limitations of our study were the small study size and the monocentric design which made it only possible to show trends in biomarker expression. A larger, multicentric validation study would be necessary before considering the use of this test to screen for children with COVID-19 or with complications such as MIS-C. Based on real-life conditions, which was one of the aims of the study, the subgroup of COVID-19 positive children was highly heterogeneous. This may have been caused by the small number, including one rare case of MIS-C with significantly different biomarker levels. A more homogenous COVID-19 cohort might have yielded more specific results.

Also of note, the score cannot identify non-infectious diseases, such as autoimmune or other disorders, whereas the expert panel was given that option in their assessment. Perhaps the confirmation rate would have been higher otherwise. Moreover, knowledge of infectious etiology is not the only factor influencing clinical decision-making, and other factors can contribute to a lack of guideline adherence [23, 24]. However, obtaining and qualitatively assessing physicians’ rationale to treat or withhold antibiotics was beyond the scope of our study.

In conclusion, the BV score showed potential to be implemented into routine diagnostics in pediatric EDs. Therefore, large prospective studies are needed to confirm our results. The goal should be to create treatment algorithms following the test’s categorizations to improve clinical practice and help counteract the spread of AMR.

## Data Availability

The datasets generated during and/or analyzed during the current study are available from the corresponding author on reasonable request.
